# Anatomy-based correction of kidney PVE on $^{177}\text{Lu}$ SPECT images

**DOI:** 10.1186/s40658-024-00612-8

**Published:** 2024-02-06

**Authors:** Julien Salvadori, Oreste Allegrini, Thomas Opsommer, Josefina Carullo, David Sarrut, Clemence Porot, Florian Ritzenthaler, Philippe Meyer, Izzie-Jacques Namer

**Affiliations:** 1 Institut de cancérologie Strasbourg Europe (ICANS), Strasbourg, France; 2 ICUBE, CNRS UMR-7357, University of Strasbourg, Strasbourg, France; 3 Université de Lyon, CREATIS, CNRS UMR5220, Inserm U1044, INSA-Lyon, Université Lyon 1, Centre Léon Bérard, Lyon, France

**Keywords:** SPECT imaging, Partial volume effect, Partial volume correction, $^{177}$Lu, Peptide receptor radionuclide therapy

## Abstract

**Background:**

In peptide receptor radionuclide therapy (PRRT), accurate quantification of kidney activity on post-treatment SPECT images paves the way for patient-specific treatment. Due to the limited spatial resolution of SPECT images, the partial volume effect (PVE) is a significant source of quantitative bias. In this study, we aimed to evaluate the performance and robustness of anatomy-based partial volume correction (PVC) algorithms to recover the accurate activity concentration of realistic kidney geometries on $^{177}$Lu SPECT images recorded under clinical conditions.

**Methods:**

Based on the CT scan data from patients, three sets of fillable kidneys with surface-to-volume (S:V) ratios ranging from 1.5 to 2.8 cm^−1^, were 3D printed and attached in a IEC phantom. Quantitative $^{177}$Lu SPECT/CT acquisitions were performed on a GE Discovery NM CT 870 DR camera for the three modified IEC phantoms and for 6 different Target-To-Background ratios (TBRs: 2, 4, 6, 8, 10, 12). Two region-based (GTM and Labbé) and five voxel-based (GTM + MTC, Labbé + MTC, GTM + RBV, Labbé + RBV and IY) methods were evaluated with this data set. Additionally, the robustness of PVC methods to Point Spread Function (PSF) discrepancies, registration mismatches and background heterogeneity was evaluated.

**Results:**

Without PVC, the average kidney RCs across all TBRs ranged from 0.66 ± 0.05 (smallest kidney) to 0.80 ± 0.03 (largest kidney). For a TBR of 12, all anatomy-based method were able to recover the kidneys activity concentration with an error < 6%. All methods result in a comparable decline in RC restoration with decreasing TBR. The Labbé method was the most robust against PSF and registration mismatches but was also the most sensitive to background heterogeneity. Among the voxel-based methods, MTC images were less uniform than RBV and IY images at the outer edge of high uptake areas (kidneys and spheres).

**Conclusion:**

Anatomy-based PVE correction allows for accurate SPECT quantification of the $^{177}$Lu activity concentration with realistic kidney geometries. Combined with recent progress in deep-learning algorithms for automatic anatomic segmentation of whole-body CT, these methods could be of particular interest for a fully automated OAR dosimetry pipeline with PVE correction.

## Background

Peptide receptor radionuclide therapy (PRRT) utilizing $^{177}$Lu-DOTATATE has become an established treatment for inoperable or metastatic neuroendocrine tumors (NETs) [1–8]. The administration of $^{177}$Lu-DOTATATE typically follows a fixed four cycle empirical schedule with a standardized injected activity of 7.4 GBq, as described in the NETTER 1 trial [5]. This scheduling approach mirrors chemotherapy, basing decisions on traditional “dose” escalation studies and establishing acceptable toxicity probabilities for major organs-at-risk (OARs), namely the kidneys and bone marrow [9, 10]. However, recent studies have shown that this approach leads to kidney absorbed dose generally below the dose limits derived from external radiotherapy (EBRT), and with large inter-patient differences [11–14]. Moreover, the moderate objective response rate observed in the NETTER 1 trial [5] and the low incidence of severe toxicity [5, 13] suggest that many patients are undertreated with this standardized regimen. As a result, significant efforts have been directed toward personalized dosimetry to individualize PRRT. Ongoing controlled clinical trials aim to demonstrate the superiority of dosimetry-based PRRT over standard dose regimens [15, 16].

In PRRT, personalized dosimetry can be achieved by administering an activity level tailored to reach the absorbed dose threshold for the kidneys [11, 14]. Typically, the maximum tolerated absorbed dose for kidneys is set at 23 Gy, aligning with a 5% probability of nephrotoxicity 5 years post-EBRT [17]. However, accumulating evidence suggests that this threshold may not be applicable to radionuclide therapy. Several studies have reported minimal nephrotoxicity with an annual loss of kidney function around 3.4% when using $^{177}$Lu-DOTATATE [5, 10, 13]. One study even reports an absorbed dose of 40 Gy administered in 8 cures without significant renal toxicity [14]. Furthermore, a clear correlation between kidney absorbed dose and nephrotoxicity has not been consistently established. Such difficulties in establishing both a clear dose–response relationship and reliable thresholds for monitoring PRRT limit the implementation of personalized dosimetry in clinical routine and may be related, at least in part, to uncertainty in the kidney dosimetry.

A key aspect of kidney dosimetry is determining the time activity curve (TAC) through sequential quantitative SPECT/CT imaging. The inherent low spatial resolution of SPECT images introduces a pronounced quantitative bias, known as the partial volume effect (PVE). For activity quantification in a volume of interest (VOI), the PVE can be considered as an exchange between different compartments (spill-over), characterized by outgoing activity (spill-out) and incoming activity (spill-in). This PVE-induced underestimation of renal activity on SPECT images can range from 25% to 50%, as demonstrated in a study using 3D-printed anthropomorphic kidneys [18].

Several methods have been proposed for partial volume correction (PVC) in both PET and SPECT imaging [19, 20]. The simplest approach uses experimentally determined recovery coefficients (RC). However, PVC using RCs may be insufficient, since they are influenced by factors other than size, including shape, object-background contrast, and position in the image. Some techniques are purely data-driven, incorporating Point Spread Function (PSF) modeling into the reconstruction algorithm [21, 22] or applying PSF deconvolution to reconstructed images [23]. Although such algorithms are now commonly available for PET and/or SPECT, they can produce Gibbs artifacts, and their efficiency remains constrained. Another strategy uses anatomical information from other imaging modalities like magnetic resonance imaging (MRI) or computed tomography (CT). In this approach, high-resolution images (from CT or MRI) are segmented into anatomically distinct VOI, and the high-frequency information extracted is used for PVC. While some methods are VOI-centric, offering only PV-corrected activity values for each segmented region, others are voxel-based and provide PV-corrected images. A majority of these anatomy-based PVC techniques assume a position-invariant PSF and a uniform distribution of activity within all segmented VOIs. This is particularly problematic in SPECT, where spatial resolution heavily depends on the camera’s distance. Both VOI and voxel-based PVC methods developed specifically for SPECT consider the position-variant PSF [24–26].

Many dosimetric studies in PRRT either neglect the quantitative bias associated with kidney PVE [12, 13, 27] or minimize this effect by measuring kidney activity concentration within a small spherical VOI [14, 28–30]. Some studies use RCs, determined experimentally using differently sized spheres, to extrapolate and compute the kidney’s RC correction factor [31–34]. Originally, anatomy-based PVC algorithms were introduced for brain PET imaging to correct spill-over between gray and white matter as well as cerebrospinal fluid [35]. While these methods have shown good results in both PET [20] and SPECT [36] brain imaging, their application to whole-body imaging has been restricted due to the time-intensive anatomical segmentation process. However, the recent advent of effective deep-learning algorithms for the automatic anatomical segmentation of whole-body CT offers a potential solution [37, 38]. These algorithms pave the way for a fully automated dosimetry pipeline for OARs, including organ segmentation for all SPECT-CT time points combined with anatomy-based PVC.

In this context, our study aims to evaluate and compare the performance and robustness of anatomy-based PVC algorithms in recovering the accurate activity concentration of realistic kidney geometries on $^{177}$Lu SPECT images recorded under clinical conditions.

## Material and methods

### Patient-based kidney phantoms

**Fig. 1:**
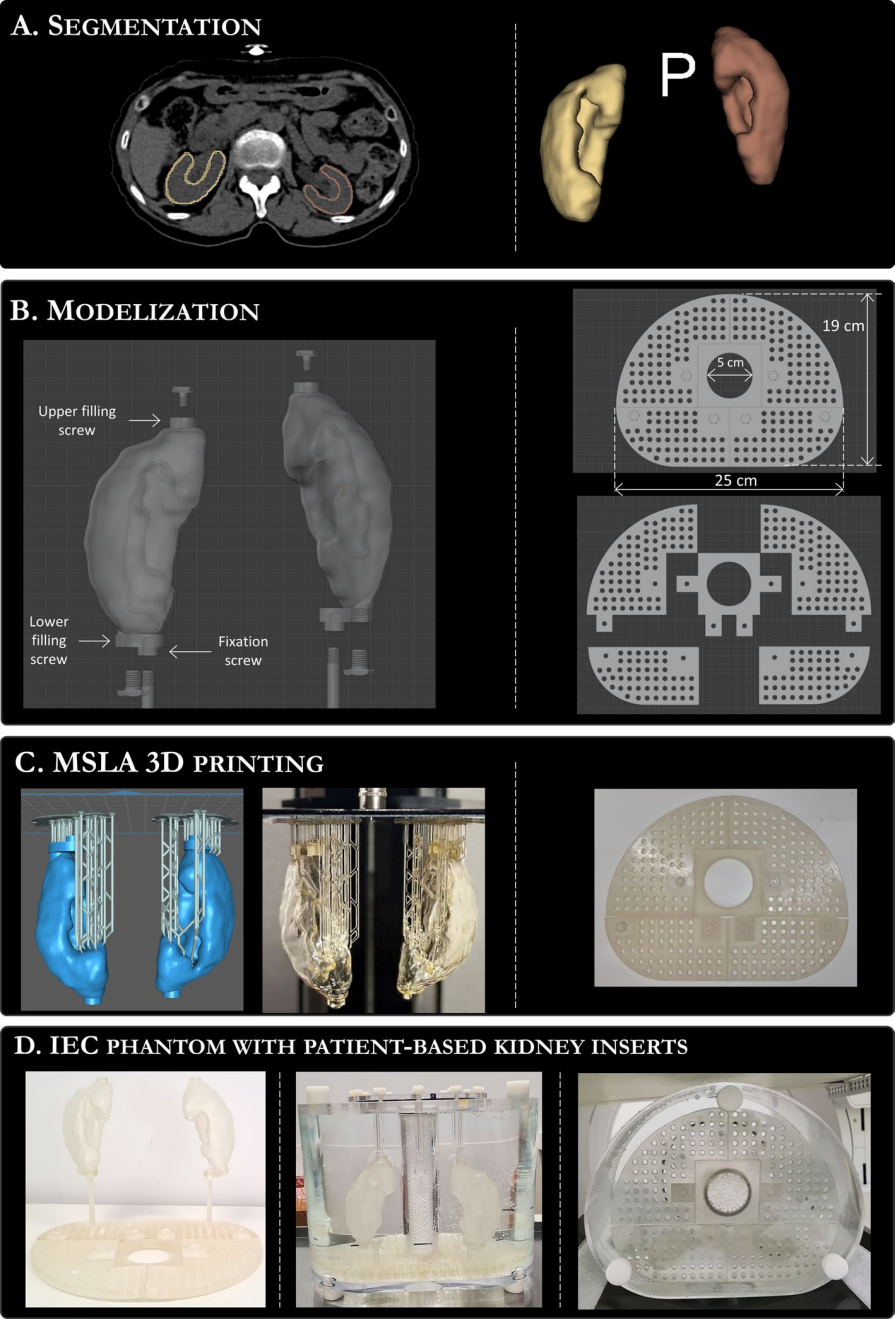
Illustration of kidney insert design and conception.** A** Manual segmentation of three kidney pairs from CT scans of patients referred for $^{68}$Ga-DOTATOC PET/CT examination (3D-Slicer).** B** Modeling of a kidney filling and fixation system, along with a modular support tailored for the IEC phantom (Blender).** C** MSLA 3D printing of kidney inserts and associated fixation system using CHITUBOX slicer software and ELEGOO SATURN printer.** D** Final assembly of the IEC phantom incorporating the patient-derived kidney inserts

Kidney phantoms were 3D-printed using CT images from $^{68}$Ga-DOTATOC PET/CT exams at the Institute of Cancerology Strasbourg Europe (ICANS) for midgut NET patients potentially eligible for PRRT. Based on 60 randomly selected examinations, 3 sizes of kidney pairs were selected (small, medium and large). The various steps for the kidney inserts conception (acquisition, segmentation, modeling and 3D-printing) are detailed in this section and illustrated in Fig. 1.

#### CT acquisition and reconstruction parameters

CT acquisitions were performed on the Vision 600 PET/CT system, featuring automatic kV selection (CARE kV), a pitch of 0.8, and adaptive mA settings (CARE Dose4D).CT images were reconstructed using Sinogram Affirmed Iterative Reconstruction (SAFIRE) with a weighting of 3, a voxel size of 0.97 × 0.97 × 2 mm, and subsequently processed with the I30f convolution kernel.

#### Segmentation and design

The three kidneys pairs were manually segmented from patients’ CT data using the 3D-Slicer software (Version 4.11.20210226, see Fig. 1A). Segmented kidneys encompass a single compartment enclosing the complete parenchyma (medulla + cortex). Kidney walls were extracted as uniform shells with a thickness of 1.5 mm using the *Hollow* tool. Table 1 lists the volumes, surfaces and surface-to-volume (S:V) ratio of each compartment surrounded by the shell.

The segmented kidney walls were exported as *.obj* file format and imported into Blender (Version 2.93.5, see Fig. 1B) a computer aided design (CAD) software. In Blender, a solid elliptical base was added to the bottom of each kidney phantom to include a screw fixation thread. Two screw filling threads were added: one at the top for filling and one at the bottom for emptying. To fix the kidney inserts inside the IEC phantom, a specific holder was modeled. The holder was a plate, 10 mm thick, shaped like the IEC phantom (scaled to 0.85 in the transverse plane) and extruded in its center to allow fixation to the lung insert. The holder consists of five distinct parts with a screw fixation system for assembly inside the IEC phantom. Fixation screw threads were designed on the entire available surface of the holder (1 thread/cm$^{2}$) and rods of different heights were modeled to fix the kidney inserts to the holder. The large number of mounting points on the holder, combined with the rods of different lengths, makes this fixation system efficient and versatile.

**Table 1:** Volume, surface and surface-to-volume ratio of each segmented kidney pair

Kidney phantom	Volume (cm$^{3}$)	Surface (cm$^{2}$)	Surface-to-volume (cm$^{-1}$)
Small-R	56.3	156.3	2.8
Small-L	70.7	163.2	2.3
Medium-R	111.6	194.7	1.7
Medium-L	113.1	201.2	1.8
Large-R	159.1	239.3	1.5
Large-L	161.0	251.3	1.6

#### MSLA 3D printing

Kidney and holder models were 3D printed using masked stereolithography (MSLA) 3D printers (SATURN, ELEGOO). MSLA uses an LCD screen to selectively block UV light from a LED array. A vat of photosensitive resin is placed above the LCD, separated by a thin fluorinated ethylene propylene (FEP) plastic layer. The UV radiant image selectively cures a resin layer on a build plate. A stepper motor moves the build plate, separating the first layer from the FEP, then descends to start the next layer. Compared to traditional fused deposition modeling (FDM) technology, MSLA offers significantly higher print resolution. The transverse resolution is determined by the LCD screen’s voxel size; while, the axial resolution depends on the motor’s step accuracy, resin type, and exposure parameters. The SATURN printer uses a 4K LCD screen (3840 × 2400 pixels) with a surface area of 192 × 120 mm$^2$ (50 µm pixels) and has a maximum printing height of 200 mm.

After modeling in Blender, all elements were exported as *.stl* file format and imported into a 3D printing slicer software (CHITUBOX V1.9.4) to design the support structures, adjust the printing parameters, and produce a series of 2D images corresponding to each layer to be polymerized. 3D prints were performed with a layer thickness of 50 microns (isotropic print resolution) using a translucent resin with a density similar to water (1.19 g/cm$^3$) and good mechanical properties (ABS-like ELEGOO translucent resin). The pieces were then washed with isopropyl alcohol (IPA) to remove excess sticky resin on the surfaces and post-cured for 5 minutes with uniform UV exposure (405 nm wavelength) to finalize polymerization and achieve full mechanical properties.

The MSLA printer specifications, resin properties, and printing parameters are detailed in Table 2.

**Table 2:** Specifications of the Saturn printers and ABS-like translucent resin with the printing parameters used in this study

ELEGOO Saturn	
LCD screen résolution (X × Y)	3840 × 2400 pixels
LCD screen dimension (X × Y)	192 × 120 mm$^2$
Maximum print height (Z)	200 mm
ABS-like ELEGOO translucent resin	
Solid density	1.195 g/cm$^3$
Flexure strength	59-70 MPa
Extension strength	36 - 53 MPa
Solidification wavelength	405 nm
Main printing parameters	
Layer height (z resolution)	50 $\upmu$m
Pixels dimension (X × Y resolution)	50 × 50 $\upmu$m$^2$
Exposure time	3.5 s
Lifting distance	10 mm

### Experimental protocol

#### Acquisition and reconstruction of fully quantitative $^{177}$Lu SPECT/CT images

All acquisitions were performed on a GE Discovery NM CT 870 DR SPECT-CT camera, equipped with Medium Energy General Purpose (MEGP) collimators and $5/8''$ thick crystal, with the acquisition and reconstruction protocol used in our clinical facility for $^{177}$Lu-DOTATATE per-treatment images for dosimetric purposes.

SPECT acquisitions involved sixty 40-second projections (30 per head) acquired in both *step and shoot* and *auto-contour* mode with 128 $\times$ 128 matrix size (pixel of 4.42 mm), a photopeak energy window of 20% width centered on 208 keV and a scatter energy window of 10% width centered on 177 keV. The SPECT reconstruction uses the Ordered Subset Expectation Maximisation (OSEM) 3D algorithm. Parameters for the algorithm were: isotropic 4.42 mm voxel size, 8 iterations, 8 subsets (64 updates), and no post-reconstruction filter. All available corrections (resolution recovery, attenuation, and scatter) were applied. Attenuation correction was CT-based and the scatter correction was performed using the Dual Energy Window (DEW) technique. All reconstructed images, initially expressed in number of detected counts, were converted to $^{177}$Lu quantitative activity (Bq/mL) using an experimentally determined Calibration Factor (CF, refer to Section “SPECT calibration and PSF characterization”), following the MIRD pamphlet 26 [33].

The CT component of the SPECT/CT imaging was performed using a low-dose protocol with a tube voltage of 100 kV, a pitch of 1.375, and an adaptive mA setting based on a noise index of 15. The images were reconstructed using 40% Adaptive Statistical Iterative Reconstruction (ASIR) with a voxel size of 0.97 × 0.97 × 2.5 mm.

#### SPECT calibration and PSF characterization

Quantitative SPECT imaging and system’s PSF characterization are mandatory to perform and evaluate anatomy-based PVC algorithms. The CF (expressed in counts/s/MBq) was determined for different levels of activity concentrations to evaluate potential dead time influences and low count rate behavior. A cylindrical phantom (internal diameter: 210 mm; length: 183 mm - Jaszczak phantom without resolution insert and spheres) was filled with 848.3 MBq (value at scan start) of a homogeneous $^{177}$Lu-DOTATATE solution and acquired eight times over 3 week. For each acquisition, the CF was determined on reconstructed images using $CF = \frac{N_\text{ave}}{C_\text{cal}}$ with $C_\text{cal}$ the calibrated activity concentration and $N_\text{ave}$ the average pixel value in a cylindrical region of interest of diameter 168 mm and length 146 mm (80% of internal Jaszczak’s dimensions), centered on the phantom. The average CF over the eight acquisitions was then used for the absolute quantification of all other SPECT images within this study.

The system’s PSF was characterized using a 3D printed capillary (50 mm length and 1.5 mm internal diameter) filled with approximately 740 GBq/L of $^{177}$Lu. The capillary was fixed in the center of the cylindrical phantom, perpendicular to the revolution axis. The phantom, filled with cold water, was then aligned and centered in the field of view. Two SPECT/CT acquisitions were performed: first with the capillary parallel to the axial x axis (for y and z resolutions) and then after a 90$^{\circ }$ rotation of the phantom along the z axis (for x resolution). From the reconstructed images, the PSF along each direction was determined as the full width at half maximum (FWHM) of a 40 mm length profile, with a 13.26 $\times$ 13.26 mm$^2$ cross section (3 × 3 voxels), going perpendicularly through the capillary’s center.

#### Kidney phantom acquisitions for various $^{177}$Lu contrasts

Three modified International Electrotechnical Commission (IEC) phantoms were prepared, integrating the holder and pairs of printed kidneys. Each phantom was initially filled with approximately 33 MBq/L of $^{177}$Lu-DOTATATE in the background and a $\sim$12 fold higher activity concentration in the target compartments which include the six spheres and both kidneys. After the first SPECT-CT acquisitions, five additional ones were performed with decreasing Target-to-Background Ratios (TBRs) of 10, 8, 6, 4, and 2 ($TBR\_i$ for $i=[12, 10, 8, 6, 4, 2]$). These TBR values were obtained by progressively adding activity to the phantom’s background. Table 3 summarizes the background and target activity concentrations with associated TBR values for the 6 acquisitions of each IEC phantom.

**Table 3:** Background and target (spheres + kidneys) activity concentrations at the start of each acquisition with associated TBR

Phantom	Activity concentration (MBq/L)		TBR
	Background	Targets (spheres + Kidneys)	
IEC 1 6 spheres + Small Kidneys	33.8	378.7	11.2
	39.7	375.8	9.5
	49.9	374.9	7.5
	65.1	373.8	5.7
	96.1	372.7	3.9
	190.2	371.1	2.0
IEC 2 6 spheres + Medium Kidneys	29.6	354.8	12.0
	34.8	352.9	10.1
	43.6	351.7	8.1
	58.6	350.9	6.0
	88.2	349.9	4.0
	171.7	347.7	2.0
IEC 3 6 spheres + Large Kidneys	32.4	357.5	11.0
	38.4	356.6	9.3
	48.2	355.6	7.4
	62.1	354.8	5.7
	104.4	354.1	3.4
	192.1	353.3	1.8

### Performance evaluation of anatomy-based PVC methods

Two VOI-based methods [Geometric Transfert Matrix (GTM) and Labbé] and five voxel-based methods [GTM + Multi-Target Correction (MTC), Labbé + MTC, GTM + Region-Based Voxel-wise correction (RBV), Labbé + RBV and Iterative Yang (IY)] were evaluated. Voxel-based PVC methods produce PV-corrected images; while, region-based PVC methods yield only PV-corrected activity values for each segmented region. All correction methods were performed using the open source PETPVC toolkit described in Thomas *et al.* [39]. The underlying principles of these techniques are summarized in the upcoming subsection (Section “Anatomy-based PVC algorithm”), followed by the methods and metrics utilized to evaluate their effectiveness in accurately determining the $^{177}$Lu activity concentration within the 6 spheres and within the three pairs of kidney inserts across a spectrum of TBR values (Section “PVC of SPECT images”).

#### Anatomy-based PVC algorithm

Both GTM ([35]) and Labbé [40, 41] are region-based methods that use piece-wise anatomical segmentation to correct multiple regions simultaneously. In these methods, the average activity of N segmented regions is stored in a vector $A = [A_{1},...,A_{N}]$ and the inverse of a matrix $G_{i,j}$ of size N $\times$ N, containing the contributions from regions *i* to regions *j* (spill-over), yields the vector $C = [C_{1},...,C_{N}]$ containing the corrected PVE values: $C = G^{-1} A$. These two methods assume uniform activity in each region but differ in the way that the $G_{i,j}$ coefficients and uncorrected values *A* are obtained. For the GTM method, the uncorrected mean value of region *i* ($A_{i}$) is computed over the exact segmentation mask $s_{i}$ and the contribution to another region *j* ($G_{i,j}$) is assessed by convolving $s_{i}$ with the PSF, applying $s_{j}$ to the resultant image, and computing the ratio between the sum of the residual voxels and the sum of $s_{j}$. In contrast, the Labbé method calculates uncorrected mean values over $s_{i}\circledast h$ and both $s_{i}$ and $s_{j}$ are convolved with the PSF to determine the $G_{i,j}$ contribution. This leads to the following equations:

1 $$\begin{aligned}{} & {} {\text{GTM}} \left\{ \begin{array}{ll} G_{i,j}= \frac{1}{\displaystyle \sum _{\text{Voxels}}{s_{j}}}\displaystyle \sum _{\text{Voxels}}{(s_{i}\circledast h)\cdot s_{j}}\\ A_{i}= \frac{1}{\displaystyle \sum _{\text{Voxels}}{s_{i}}}\displaystyle \sum _{\text{Voxels}}{s_{i}\cdot I} \end{array} \right. \end{aligned}$$

2 $$\begin{aligned}{} & {} Labb\acute{e} \left\{ \begin{array}{ll} G_{i,j}= \frac{1}{\displaystyle \sum _{\text{Voxels}}{s_{j}}}\displaystyle \sum _{\text{Voxels}}{(s_{i}\circledast h)\cdot (s_{j}\circledast h)}\\ A_{i}= \frac{1}{\displaystyle \sum _{\text{Voxels}}{s_{i}}}\displaystyle \sum _{\text{Voxels}}{(s_{i}\circledast h)\cdot I} \end{array} \right. \end{aligned}$$

With *I* the uncorrected images, *h* the system PSF, $\Sigma$ the sum over voxels values and $\circledast$ the 3D convolution operator.

Both Multi-Target Correction (MTC, [42]) and Region-Based Voxel-wise correction (RBV, [43]) are voxel-based methods and require as input the corrected activity distribution (vector $C = [C_{1},...,C_{N}]$), typically pre-computed using GTM or Labbé. The MTC method adopts a sequential target-neighbor correction approach. In this method, each region is successively treated as a target and corrected for PVE with adjacent regions:

3 $$\begin{aligned} I_{c}=\sum _{j}\left( \frac{s_{j}\cdot I}{s_{j}\circledast h}-\frac{s_{j}\cdot \sum _{i\ne j}(C_{i}\cdot s_{i})\circledast h}{s_{j}\circledast h}\right) \end{aligned}$$

where $I_{c}$ is the PV-corrected image and *I* the observed image. This equation shows that MTC uses the image to correct for spill-out (left side) and uses the pre-computed corrected activity distribution to correct for spill-in (right side).

In contrast to MTC, RBV corrects simultaneously all regions by generating a piece-wise constant image using the GTM or Labbé pre-computed values $S=\sum \limits _{j} C_{j}\cdot s_{j}$ and applying the following voxel-wise correction:

4 $$\begin{aligned} I_{c}=I\cdot \frac{S}{S\circledast h} \end{aligned}$$

As a consequence, spill-out and spill-in are simultaneously computed for each region using only the pre-computed corrected activity distribution.

The Iterative Yang (IY, [20]) technique is a voxel-based method similar to the RBV one but does not require any prior information on the corrected activity values. A first iteration is performed using equation (4) with uncorrected mean values estimated directly from the SPECT image *I*. The mean values are then extracted from corrected images and provided to the algorithm for a second iteration. The process is iterated until the corrected mean values converge. In this study, 10 iterations was used as suggested by Thomas et al [39].

#### PVC of SPECT images

Within 3D-Slicer, 11 structures were manually segmented for each IEC phantom: the two kidneys, the six spheres, the lung insert, the holder and the background. Segmentations were performed on the CT images corresponding to a TBR of 12 ($CT_{TBR\_12}$) and were exported in RTSTRUCT format. Subsequent post-processing was executed with MATLAB (MATLAB R2021a, academic license).

All SPECT images were resampled with nearest neighbors interpolation to isotropic voxels of 2.21 mm. RTSTRUCTs of the $CT_{TBR\_12}$ were imported and converted to mask before being resampled (*nearest* method) with 2.21 mm voxels and aligned to the corresponding SPECT image ($SPECT_{TBR\_12}$). All $CT_{TBR\_i}$ were rigidly registered to $CT_{TBR\_12}$ and the obtained transformation matrix was used to propagate all SPECT segmentations from the $SPECT_{TBR\_12}$ image to the SPECT images corresponding to the other TBRs. For the 3 phantoms and the 6 TBRs, the quantitative SPECT images (in Bq/mL), the anatomical segmented ROIs, and the experimentally derived PSF (see Section “SPECT calibration and PSF characterization”) were provided as input to the different PVC correction algorithms (PETPVC toolkit [39]).

**Fig. 2:**
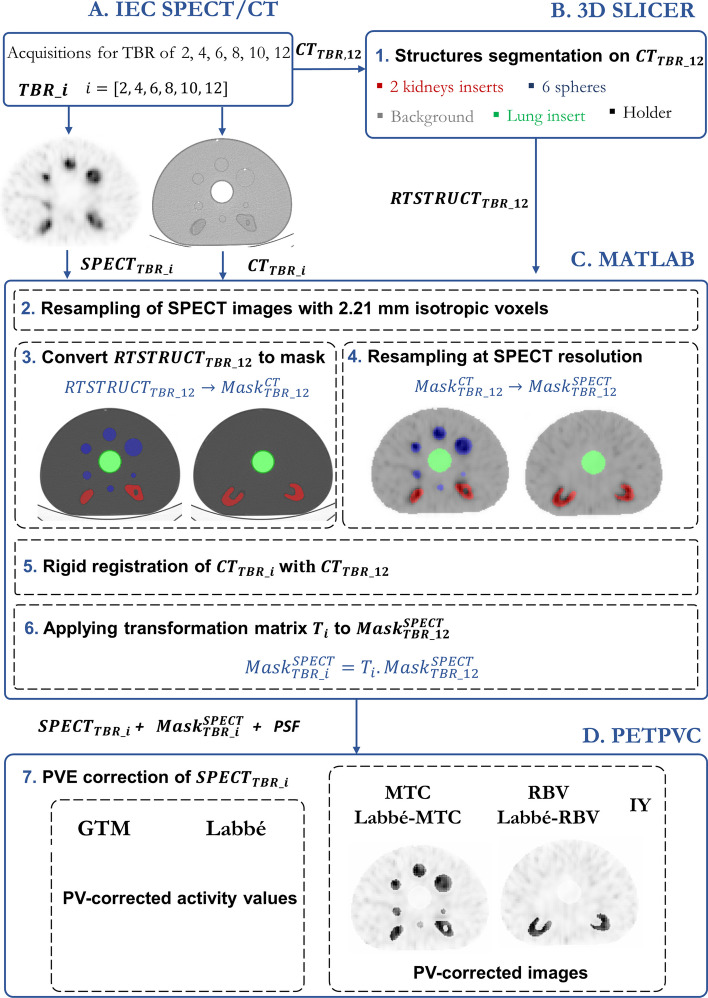
Illustration of the procedure used to evaluate the seven PVC algorithms.** A** Fully quantitative SPECT-CT acquisitions of the 3 modified IEC phantoms for 6 TBR values.** B** Manual segmentation of structures using 3D-Slicer on the CT images corresponding to a TBR of 12 ($CT_{TBR\_12}$). The segmented structures are then exported in RTSTRUCT format ($RTSTRUCT_{TBR\_12}$).** C** Within MATLAB, $RTSTRUCT_{TBR\_12}$ is converted into a labeled mask at SPECT resolution ($Mask^{SPECT}_{TBR\_12}$). This mask is rigidly propagated onto the masks corresponding to the other TBRs ($Mask^{SPECT}_{TBR\_i}$) using the transformations derived from the alignment of $CT_{TBR\_12}$ with $CT_{TBR\_i}$** D** Fully quantitative SPECT recordings ($SPECT_{TBR\_i}$), structure masks ($Mask^{SPECT}_{TBR\_i}$), and camera PSF serve as inputs to the PETPVC software

The different steps of the used method (segmentation, registration, propagation and correction) are summarized in Fig. 2.

#### Recovery coefficient calculation

The ability to recover the real activity concentration in the target regions (2 kidneys and 6 spheres) was assessed using the recovery coefficients $RC = \frac{C}{C_{\text{cal}}}$, where *C* is the average activity concentration extracted from the anatomical segmentation used to perform PVE corrections and $C_{\text{cal}}$ is the corresponding calibrated activity concentration. To control the phantom preparation, the camera calibration factor and the absence of dead time effects, a RC was also calculated in the homogeneous region of the background by averaging the activity concentration in a VOI eroded by 3 cm with respect to the initial background segmentation.

### Robustness evaluation of anatomy-based PVC methods

Because all evaluated PVC methods rely on PSF, registration and uniform activity, we assessed their sensibility and robustness to PSF discrepancies, registration mismatches and background heterogeneity.

#### PSF and registration mismatch

The influence of PSF discrepancy on kidney PVC was evaluated for a TBR of 12 by performing PVC using all methods with underestimated and overestimated PSF values ($\pm$ 6 mm in all directions, 2 mm step). Additionally, the effect of misalignment between the segmented and real structures, attributed to imprecise SPECT-CT rigid registration, was also examined with a TBR of 12 by translating the kidney mask along each direction ($\pm$ 3 voxels in all directions, 1 voxel step).

#### Background heterogeneity

Heterogeneity in the background compartment was evaluated from the images corresponding to a TBR of 12 by including the lung insert segmentation in the background segmentation and by artificially and uniformly adding activity in the voxels within the lung insert. The resulting heterogeneity is sufficiently distant from the target regions to avoid PVE influence and could thus mimic situations like the absence of bladder or liver segmentation on post-treatment $^{177}$Lu-DOTATATE images. The intensity magnitude was gauged by the heterogeneity ratio, defined as the quotient of average activities in the background compartment with and without the presence of the heterogeneity.

The influence of background heterogeneity on kidney PVC were evaluated by performing PVE correction using all PVC methods and escalating values of heterogeneity ratios (ranging from 1 to 4, 0.25 step)

## Results

### SPECT calibration and PSF characterization

**Fig. 3:**
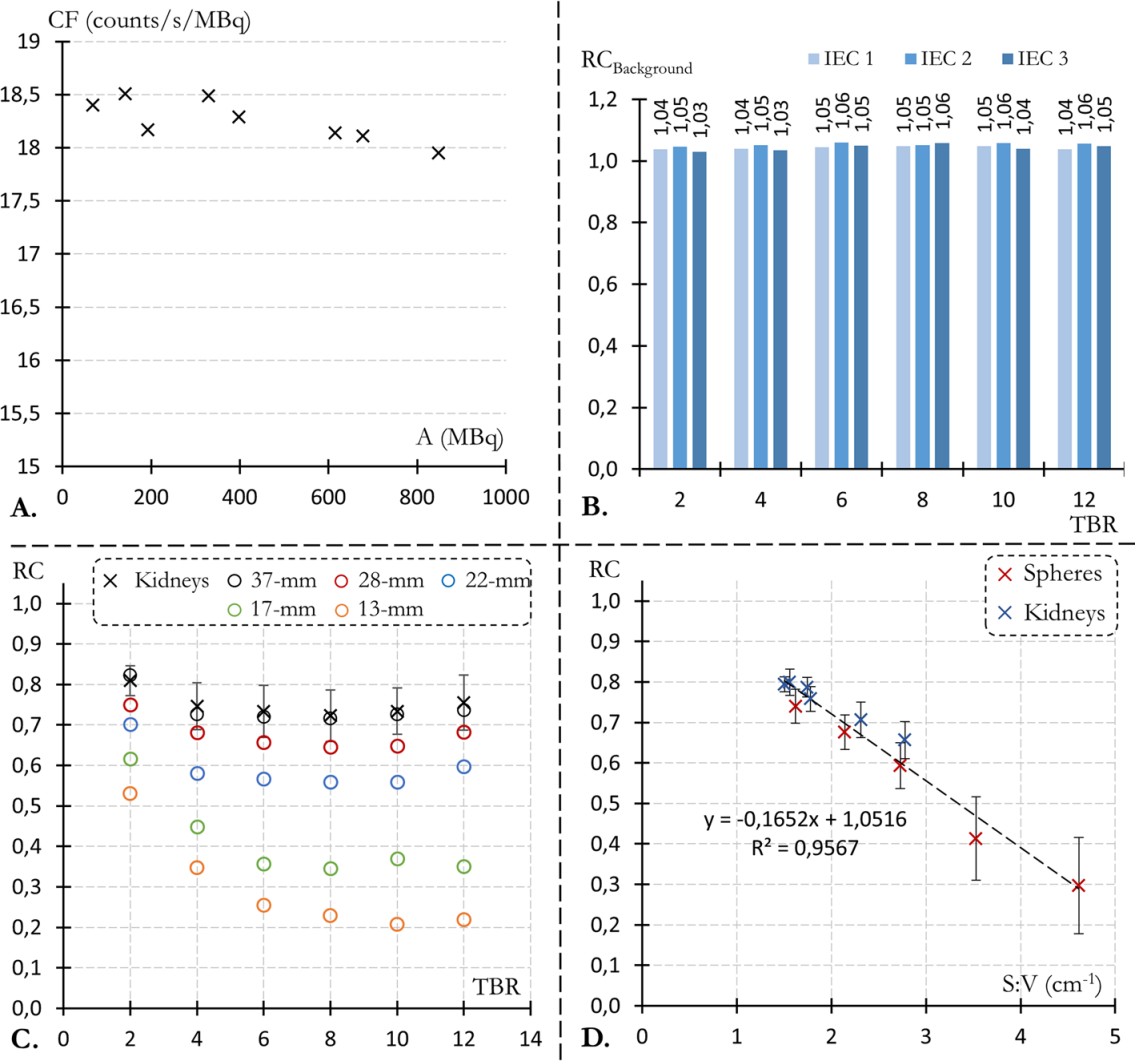
**A** Tomographic calibration factors derived from acquisitions of a homogeneous $^{177}$Lu-DOTATATE Jaszczak phantom over a wide range of activity values, using our clinical recording and reconstruction protocol for $^{177}$Lu-dosimetry.** B** Background RC, computed within a region eroded by 3 cm from the original background segmentation, for the 18 measurements of the modified IEC phantom (3 kidney pairs and 6 TBRs).** C** Uncorrected RC plotted against TBR for the kidneys (black crosses, mean and std over the 6 kidney inserts) and for spheres ranging in size from 13-mm (orange circles) to 37-mm in diameter (black circles).** D** Uncorrected RC plotted against S:V ratio for the 6 kidney inserts (blue symbols, mean and std over the 6 TBRs) and the 5 spheres (red symbols, mean and std over the 18 recordings), complemented by the associated linear regression

As depicted in Fig. 3A, the tomographic calibration factor was approximately constant over the wide range of $^{177}$Lu activity tested (between 68 and 848 MBq) with 18.26 ± 0.20 ($\sigma$) counts/s/MBq. CF remains stable for small activities, at very low count statistics. However, a slight degradation was observed for the highest activities with a CF decrease of 2.45% between the recordings performed at 68.1 MBq and 848 MBq (18.40 vs. 17.95 counts/s/MBq). This degradation can be attributed to count losses from saturation effects, including dead time and pile-up integration time.

The PSF at the FOV center was 8.8, 11.0 and 9.2 mm (FWHM) along the x, y and z directions, respectively. The anisotropy observed in the transverse (XY) plane arises from the “auto-contour” mode and the radial dependence of the spatial resolution. Given the table + phantom (or table + patient) dimensions, the detectors’ elliptical rotation has its major axis along the x-axis (left-right) and minor axis along the y-axis (anterior-posterior), yielding better resolution in the x direction.

### Performance of anatomy-based PVC methods

#### Uncorrected RC

For the 18 acquisitions (3 kidney inserts for 6 TBRs), background was accurately quantified with RCs ranged from 1.03 to 1.06 (1.05 ± 0.01, Fig. 3B). RCs for the kidneys and the larger spheres (those exceeding 17 mm in diameter) remained relatively TBR-independent for values between TBR 4 and 12, with a maximum relative difference ranging from 6.9% (22 mm sphere) to 4.4% (kidney) (Fig. 3C). Additionally, the average kidney RCs across all TBRs ranged from 0.66 ± 0.05 to 0.80 ± 0.03 (Fig. 3D) and the average spheres RCs varied from 0.30 ± 0.12 (13 mm diameter) to 0.74 ± 0.04 (37 mm diameter). The 10 mm sphere was not reported as it was indistinguishable from the background noise for all TBRs. Lastly, both spheres and kidney RCs exhibited a linear correlation with the S:V ratio (Fig. 3D).

#### PVC RC

**Fig. 4:**
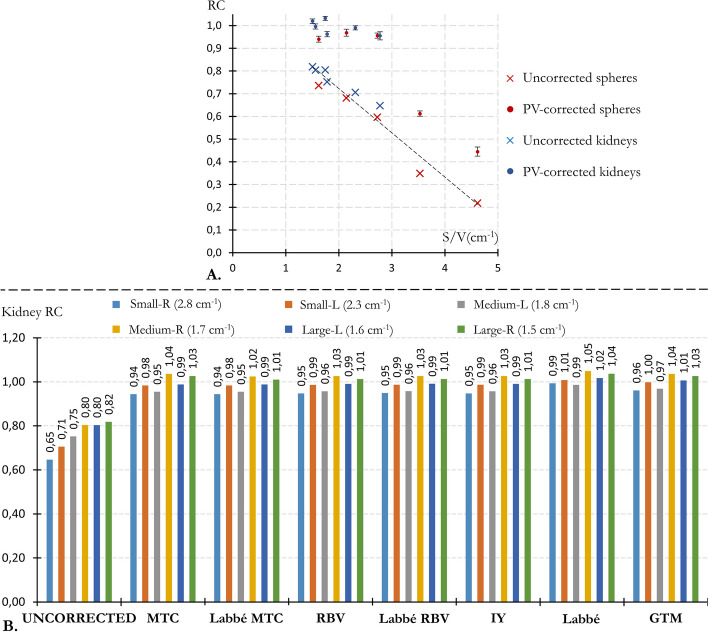
PVC results for a TBR of 12.** A** Uncorrected RCs (cross symbols) and PV-corrected RCs (point symbols, mean and std over the 7 PVC methods) plotted against the S:V ratio, for both the 6 kidney inserts (blue symbols) and the 5 spheres (red symbols).** B** Kidney RCs before and after PVC

At a TBR of 12, all anatomy-based PVC method were able to recover the kidneys activity concentration with an error below 6% (with RCs between 0.94 and 1.05, illustrated in Fig. 4). Moreover, Fig. 4A shows that only the spheres of 13 mm and 17 mm diameter were not effectively PVE corrected, with a mean corrected RC across all method far below 0.9 (standing at 0.43 and 0.61, respectively).

**Fig. 5:**
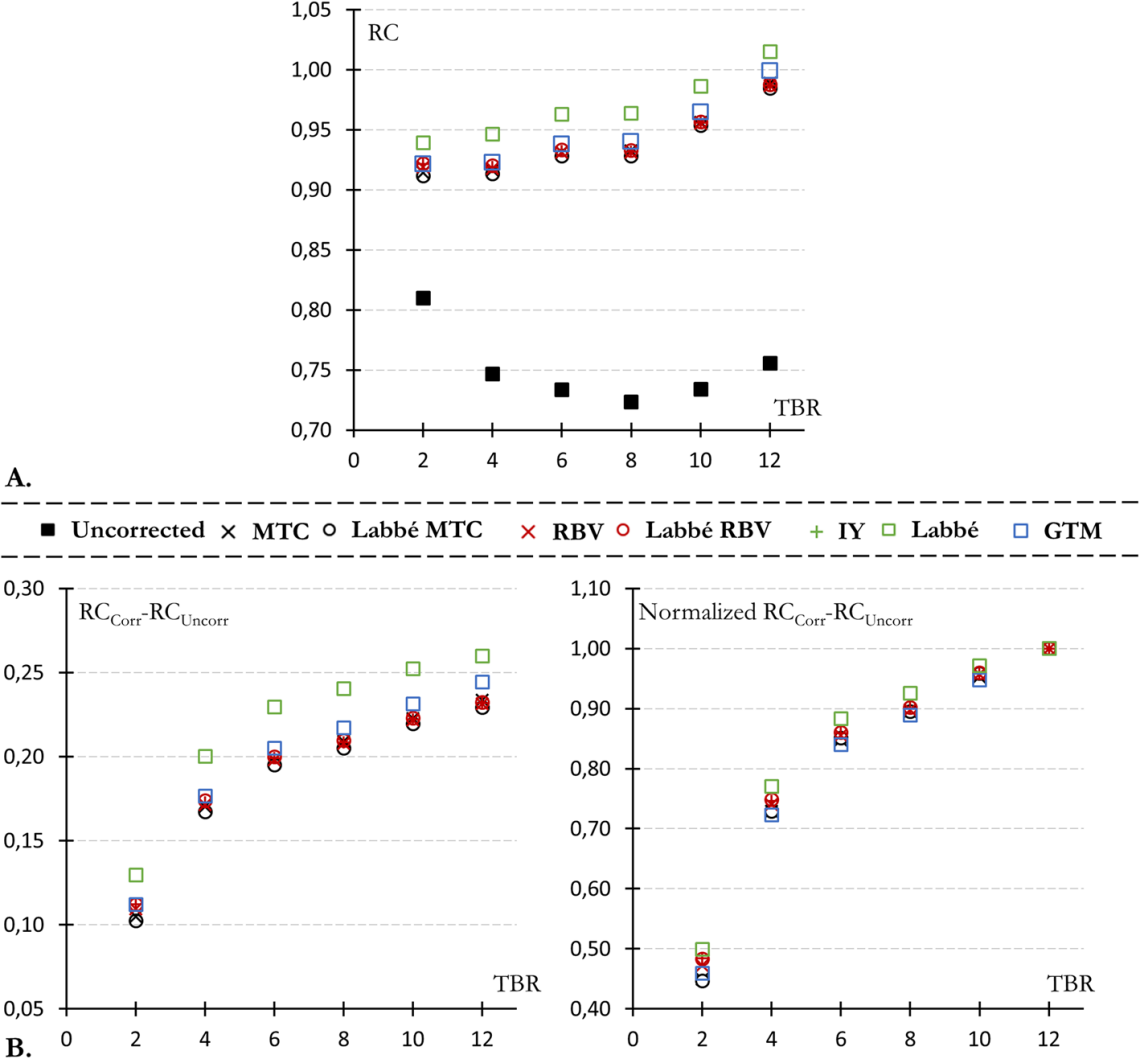
**A** Uncorrected (black solid squares) and corrected (empty squares) RC (averaged over the 6 kidneys) plotted against the TBR.** B** Relationship between the recovery of kidney RC (averaged over the 6 kidneys) and TBR, displayed with normalization by the highest TBR value (TBR_12) in the right panel and without normalization in the left panel

Most PVC methods yielded similar results, with the exception of the Labbé method which overestimated kidney RCs by an average of 3% relative to other techniques (refer to Figs. 4B and 5A and B). This difference between Labbé and other methods is almost independent of the TBR (Fig. 5). Furthermore, all methods result in similar declines in RC restoration as the TBR decreases (Fig. 5B and C). Compared with a TBR of 12, the average relative loss of kidney recovery was 2.7, 8.1, 12.8, 24.6, and 50.9% for TBRs values of 10, 8, 6, 4, and 2, respectively.

**Fig. 6:**
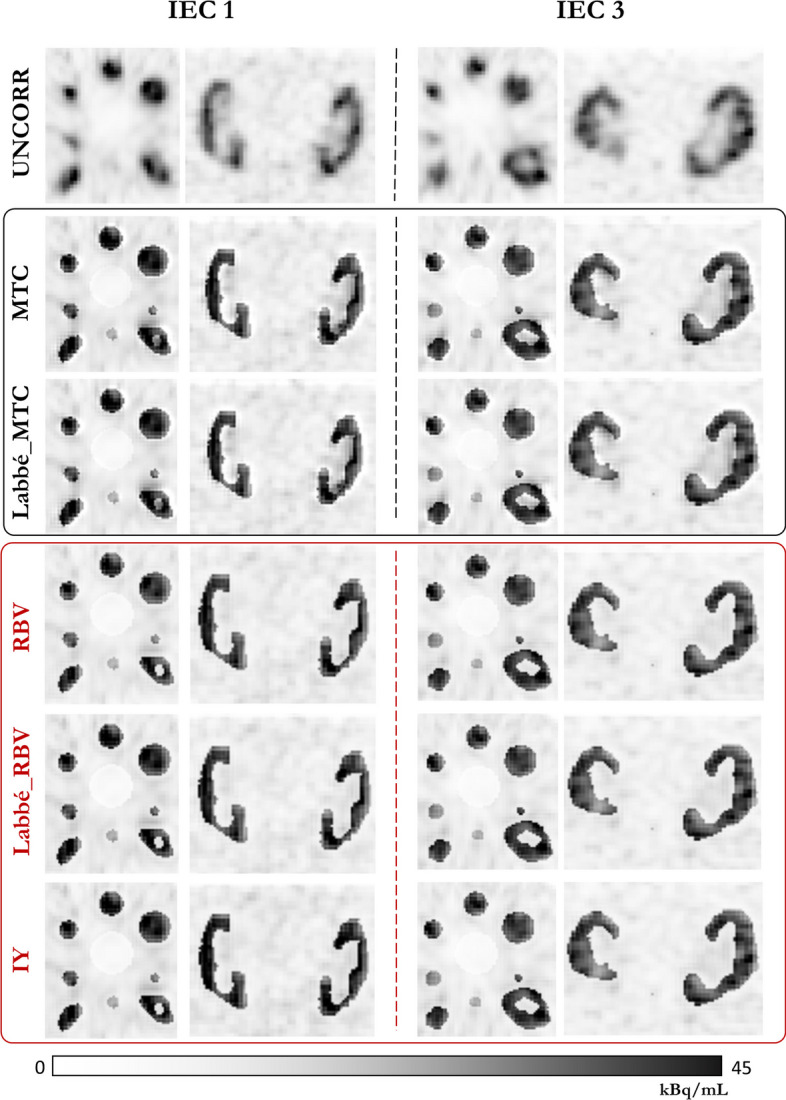
Representative example for two of the three modified IEC phantoms (IEC 1 and 3) of an axial slice passing through the center of the spheres and a coronal slice passing through the two kidneys, before (first line) and after voxel-based PVC

**Fig. 7:**
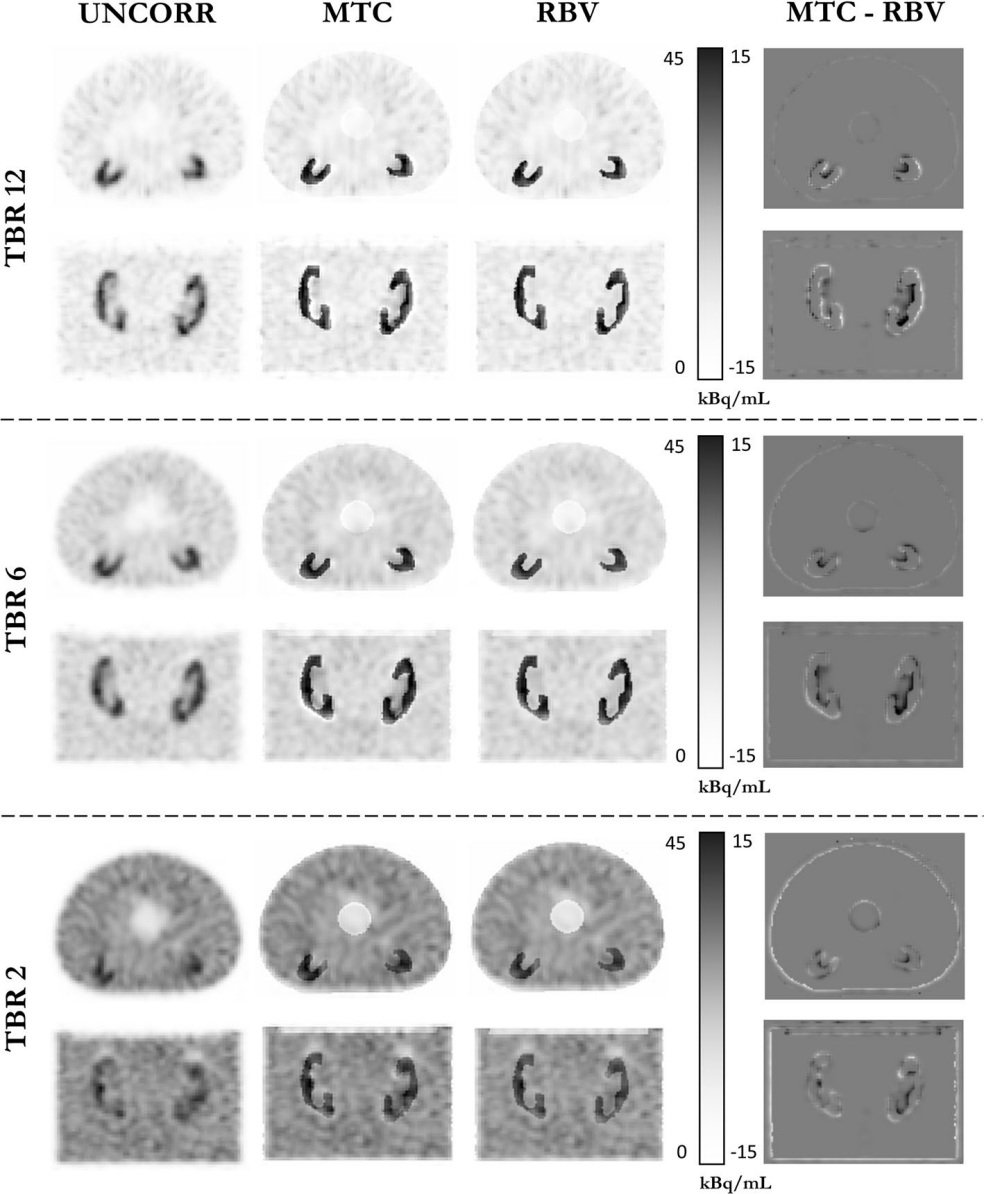
Representative examples of the IEC 1 phantom are presented for three TBR values ($TBR\_12$, $TBR\_6$, and $TBR\_2$). The images illustrate an axial slice passing through the sphere centers and a coronal slice through the two kidneys. The left column presents uncorrected images, the central columns display images after correction with MTC and RBV, and the right column depicts the voxel-to-voxel difference between MTC- and RBV-corrected images

As illustrated in Fig. 6, the choice of Labbé or GTM to precalculate corrected mean values for MTC and RBV had barely any influence on the images. Images corrected with IY were very close to those corrected with RBV. However, MTC-corrected images are less uniform than RBV-corrected images at the outer edge of high-uptake areas (kidneys, spheres). This effect, seen in Fig. 7, intensifies with increasing TBR, leading to an average MTC RC overestimation at the kidney’s edge that ranges from 1% (at TBR 2) to 8% (at TBR 12). Peripheral RCs were assessed using three-voxel-thick shells encircling the kidney masks.

### Robustness of anatomy-based PVC methods

**Fig. 8:**
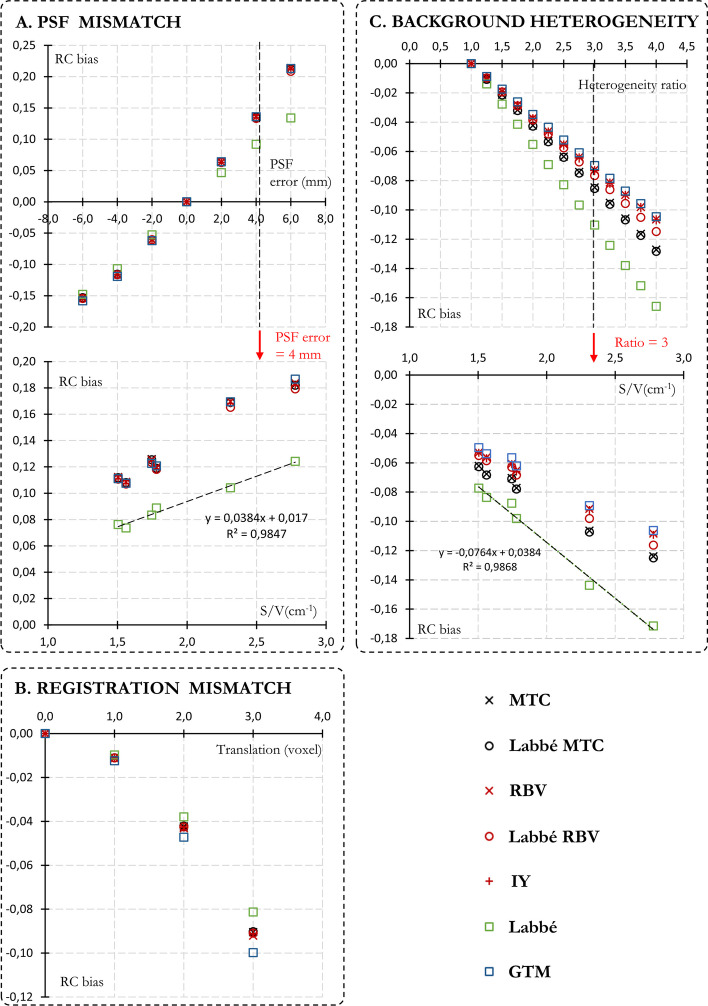
Influence of PSF mismatch (**A**), registration mismatch (**B**), and background heterogeneity (**C**) on the performances of PVC methods for a TBR of 12.** A** Kidney RC bias (averaged over the 6 kidneys) with respect to PSF mismatch (± 6 mm, 2 mm step, upper panel) and S:V ratio (4 mm PSF error, lower panel).** B** Kidney RC bias with respect to registration mismatch (1, 2 and 3 voxel sizes translations).** C** Kidney RC bias with respect to background heterogeneity (heterogeneity ratio ranging from 1 to 4 with 0.25 step, upper panel) and S:V ratio (heterogeneity ratio of 3, lower panel)

Overall, PVC methods were more susceptible to PSF errors than registration mismatches (Fig. 8A and B). PSF errors of 2, 4 and 6 mm resulted in an average absolute RC bias 514, 242 and 136% higher than offsets of the same amplitude.

As depicted in Fig. 8A, the Labbé method was the less susceptible to PSF mismatch and, particularly overestimations, with mean positive kidney RCs biases of 4.7, 9.2 and 13.4%, for PSF errors of + 2, + 4 and + 6 mm, respectively. All other methods exhibited consistent behavior with mean positive biases of 6.3, 13.5, and 21.2%. For all PVC methods, a linear correlation was observed between the bias resulting from PSF mismatch and the S:V ratio (Fig. 8A, bottom panel). The Labbé method was also the less susceptible to registration mismatch with mean positive kidney RC biases of 1.0, 3.8 and 8.1% for shifts of + 1, + 2 and + 3 voxels, respectively (refer to Fig. 8B). In contrast, GTM was the most affected with mean positive RCs bias of 1.2, 4.7 and 10%. All other voxel-based techniques showcased average positive biases that sat between the values of GTM and Labbé.

As illustrated in Fig. 8C, artificially inserting a hot, non-segmented structure into the background, at a sufficient distance from the kidneys to avoid PVE (lung insert), results in an overestimation of the subtracted spill-in (background’s contribution to the kidneys). This leads to a mean negative RC bias that linearly relies on the heterogeneity ratio (4.1, 8.2 and 12.2% for ratios of 2, 3 and 4, respectively). For all PVC methods, a linear correlation was observed between the bias resulting from background heterogeneity and the S:V ratio (Fig. 8B, bottom panel). The Labbé method is the most vulnerable to deviations from the assumption of background homogeneity. For all heterogeneity ratio, the absolute RC bias with Labbé was $\sim$43% greater than the average bias across other methods (7.7 vs. 11% for a ratio of 3).

## Discussion

In this study, we assessed the performance and robustness of seven anatomy-based PVC algorithms on three IEC phantoms featuring patient-derived kidney inserts, filled with $^{177}$Lu and recorded following our clinical protocol across six TBRs.

Overall, anatomy-based PVC methods successfully recovered the kidney activity concentration, with a mean corrected RC of 0.96 across all methodologies and TBRs. Their efficiency decreased at lower TBR, as illustrated in Fig. 5A. This behavior result from a mismatch between the TBR dependency anticipated by PVC methods and the observed constancy in uncorrected RCs. As depicted in Fig. 5B, all PVC techniques consider the expected rise in spill-in (from background to targets) as TBR decreases, by reducing the RC correction accordingly. However, this TBR-related increase in spill-in was notably absent on uncorrected kidney RCs for TBR values between 4 and 12 (Fig. 5A, solid black square), which results in the underestimation observed for low TBR values. One potential explanation for this low dependence of RCs with TBR in uncorrected images might be the regularization introduced by the PSF modeling within the reconstruction algorithm.

Without PVC, accurate background quantification was achieved in reconstructed images from the 18 recorded IEC phantoms, averaging a relative RC bias of 4.7% (Fig. 3B). This bias, which may be linked to small uncertainty over phantom preparation and camera calibration factor, was considered acceptable and no background correction factor was thus applied to the target RC (kidneys and spheres).

Customized IEC phantoms led to a reconstructed activity distribution of kidney uptake that closely mirrors an actual patient, as observed in coronal slices through the kidney insert (Figs. 6 and 7). Quantitatively, the fraction of scattered and attenuated photons may differ between a patient and the corresponding phantom. However, it is likely that these differences are effectively addressed during attenuation and scattering corrections, leading to accurate restitution of kidney RC.

Uncorrected RC ranged from 0.66 to 0.82 for kidneys and to 0.30 (13 mm) to 0.75 (37 mm) for distinguishable spheres. This underestimation of renal activity concentration by 18–34% due to PVE aligns with the observations made by Grings et al. [18].

Close results were obtained for all PVC methods, both quantitatively (Figs.  4 and  5) and visually (Fig. 6). The Labbé method provided slightly higher RCs than the other methods (3% on average). MTC-based PVC images (including MTC and Labbé-MTC) were slightly less uniform at the edges of high-uptake areas (kidneys and spheres) than RBV-based PVC methods (including RBV, Labbé-RBV and IY), with a TBR-related quantitative bias (Fig. 7). Unlike RBV, which corrects both spill-out and spill-in using a constant piece-wise image composed of the pre-computed corrected values, MTC uses sequentially the voxels of each image compartment to correct for spill-out. The potential advantage is the intrinsic consideration of target region heterogeneity when correcting for spill-out. On the other hand, this makes the method asymmetrical, since spill-in is corrected (as for RBV) with the constant piece-wise activity distribution precalculated by using Labbé or GTM. Consequently, when correcting for background spill-out, the MTC method is affected by the PVE of hot structures, shaping the heterogeneity and quantitative bias observed at the edges of kidneys and spheres. Furthermore, using Labbé instead of GTM to precalculate the corrected activity distribution as input for MTC and RBV has almost no influence, both quantitatively on RC restauration (Figs. 4B and 5) and visually on the reconstructed images (Fig. 6).

To the best of our knowledge, it is the first time that anatomy-based PVC algorithms were experimentally evaluated to improve kidney quantification in the framework of personalized PRRT dosimetry. However, it must be recognized that this phantom study was not fully representative of clinical conditions. PSF characterization was performed with an average radius of rotation close to that subsequently used on IEC phantoms, minimizing the error associated with non-stationary spatial resolution. A higher bias would be expected for corpulent patients. In addition, all SPECT-CT registrations were carefully checked, and the phantom remained perfectly rigid and static during acquisitions, which contrasts with clinical practice where patient movement, SPECT-CT misregistration, and respiratory motion can cause blurring and artifacts in the SPECT image. Finally, all structures with an uptake that differs from the background were precisely segmented to get as close as possible to the uniformity assumption, which in practice is difficult and time-consuming to be achieved clinically on $^{177}$Lu SPECT-CT images (kidneys, bone marrow, liver, lung, spleen...). Nevertheless, the present study demonstrated that anatomy-based PVC methods offer some robustness to PSF inadequacy, registration mismatch and background heterogeneity. Kidneys RC bias averaged over all methods remains below 5% for PSF inadequacy < 1.7 mm (Fig. 8A), registration errors < 4.8 mm (1.7 voxels, Fig. 8B) and heterogeneity ratio < 2.2 (i.e., average background concentration 2.2 times higher than local concentration around kidney inserts, Fig. 8C).

Interestingly, PVC methods were more sensitive to PSF mismatch than registration error with higher mean RC bias of 514% 242% and 136% for same offsets of 2, 4 and 6 mm, respectively. As shown in Equation (1) and  (2) for the GTM and Labbé PVC methods, a PSF inadequacy will mainly impact $G_{i,j}$ contributions; while, a registration error will only impact the estimated uncorrected values $A = [A_{1},...,A_{N}]$. Given the limited spatial resolution of $^{177}$Lu SPECT examinations, the quantitative bias on *A* resulting from a registration mismatch will be much more limited than the bias on $G_{i,j}$ resulting from a PSF inadequacy of the same magnitude. This last observation underlines the importance of accurately characterizing the system’s PSF when using PVC methods.

Among PVC methods, Labbé was the least sensitive to both PSF and registration mismatches, with a RC bias 27% and 11% lower than the other methods for a 4 mm PSF error and 2 voxels translations, respectively. On the other hand, the Labbé method was also the most sensitive to the violation of the background uniformity assumption with a RC bias 43% higher than the other PVC methods for all heterogeneity ratio. Compared to the GTM approach, this behavior of the Labbé method is closely related to the additional PSF convolution applied for both $A_{i}$ and $G_{i,j}$ estimation (see Eq. (1) versus Eq. (2)). In addition, the specific behavior of the Labbé method when the underlying assumptions are not satisfied was no longer observed with the Labbé-MTC and Labbé-RBV approaches. Voxel-based methods compensate for the bias introduced by the Labbé method and provide results close to those of GTM.

Consistent with the recent paper by Grings et al. [18], a linear correlation was observed between uncorrected RCs and the S:V ratio of targets regions (Fig. 3D). An additional finding of this study was that the S:V ratio not only linearly models uncorrected RCs, but also the degradation observed with PSF inadequacy (Fig. 8A) and background heterogeneity (Fig. 8C). In addition, only the two smallest spheres were not effectively PVE corrected (Fig. 4A). A resolution-related cutoff S:V ratio, above which anatomical PVC methods no longer effectively compensate for PVE, seems to be around 3 cm$^{-1}$.

This study is the first step toward a fully automated dosimetry pipeline for PRRT with anatomy-based PVC that can be implemented in a clinical environment. Consequently, PVC methods with distance-variant PSF [24–26] were not considered here, as they require access to a manufacturer-independent OSEM algorithm with detection system modeling, which is not usually available in a clinical environment. Nevertheless, these approaches developed specifically for SPECT, would probably improve both the performance and robustness of kidney PVC.

Finally, it should be pointed out that the main obstacle to implementing these methods in a clinical environment remains the time-consuming segmentation task. However, we believe that recent efforts made by the PET community to develop automatic whole-body CT segmentation algorithms to facilitate the systemic investigations of the human body via whole-body PET imaging, may be of great help to overcome this limitation [37, 38].

## Conclusion

Anatomy-based PVC methods enable accurate $^{177}$Lu quantification inside realistic kidney geometries. These methods are however sensitive to incorrect PSF modeling. The Labbé method offers the highest robustness to PSF and registration errors, but is also the most sensitive to background heterogeneity. When a VOI-based method is sufficient, the Labbé approach should be used whenever the PSF is not precisely known. Conversely, in cases of large background heterogeneity (for example due to registration or segmentation errors), the GTM approach seems more suitable. When a corrected image is required (and not only the organ-based activity), RBV-based methods (RBV, Labbé-RBV, IY) seem more appropriate due to the heterogeneities observed with the MTC method at the periphery of segmented regions with high uptake. Combined with recent advances in deep-learning based algorithms for automatic organs segmentation of whole-body CT, these approaches could be of particular interest for fully automated OAR dosimetry pipeline with PVE correction.

## Data Availability

The datasets used and/or analyzed during the current study are available from the corresponding author on reasonable request.

## Abbreviations

CAD: Computer aided design
CF: Calibration factor
CT: Computed tomography
DEW: Dual energy window
EBRT: External beam radiation therapy
FEP: Fluorinated ethylene propylene
FDM: Fused deposition modeling
FWHM: Full width at half maximum
GE: General electric
GTM: Geometric transfer matrix
ICANS: Institut de Cancerologie Strasbourg Europe
IEC: International Electrotechnical Commission
IPA: Isopropyl alcohol
IY: Iterative Yang
LCD: Liquid crystal display
MEGP: Medium energy general purpose
MRI: Magnetic resonance imaging
MSLA: Masked stereolithography
MTC: Multi-target correction
NET: Neuroendocrine tumor
OAR: Organ at risk
OSEM: Ordered subset expectation maximization
PRRT: Peptide receptor radionuclide therapy
PSF: Point spread function
PVC: Partial volume correction
PVE: Partial volume effect
RBV: Region-based voxel-wise
RC: Recovery coefficient
S:V: Surface to volume
SPECT: Single photon emission computed tomography
TAC: Time activity curve
TBR: Target to background ratio
UV: Ultraviolet
VOI: Volume of interest
ASIR: Adaptive statistical iterative reconstruction
SAFIRE: Sinogram affirmed iterative reconstruction
